# Extravasation of leukocytes in comparison to tumor cells

**DOI:** 10.1186/1478-811X-6-10

**Published:** 2008-12-04

**Authors:** Carina Strell, Frank Entschladen

**Affiliations:** 1 Institute of Immunology, Witten/Herdecke University, Stockumer Str. 10, 58448 Witten, Germany

## Abstract

The multi-step process of the emigration of cells from the blood stream through the vascular endothelium into the tissue has been termed extravasation. The extravasation of leukocytes is fairly well characterized down to the molecular level, and has been reviewed in several aspects. Comparatively little is known about the extravasation of tumor cells, which is part of the hematogenic metastasis formation. Although the steps of the process are basically the same in leukocytes and tumor cells, i.e. rolling, adhesion, transmigration (diapedesis), the molecules that are involved are different. A further important difference is that leukocyte interaction with the endothelium changes the endothelial integrity only temporarily, whereas tumor cell interaction leads to an irreversible damage of the endothelial architecture. Moreover, tumor cells utilize leukocytes for their extravasation as linkers to the endothelium. Thus, metastasis formation is indirectly susceptible to localization signals that are literally specific for the immune system. We herein compare the extravasation of leukocytes and tumor cells with regard to the involved receptors and the localization signals that direct the cells to certain organs and sites of the body.

## Introduction

### General steps of the extravasation process

The extravasation is a multi-step process of the emigration of cells from the blood stream into the tissue. The most prominent types of extravasating cells are leukocytes, i.e. T lymphocytes, natural killer (NK) cells, neutrophil granulocytes and monocytes. These cells have to leave the blood vessels in order to reach tissue sites of inflammation, infection or injury. But not only the emigrating cells, the vascular endothelium takes actively part in the extravasation process, too. It has to deliver certain localization signals, so that the leukocytes know where and when to emigrate. Therefore, the endothelium changes dynamically its architecture including receptor expression and cell-cell contacts. In contrast to the important and physiological extravasation of leukocytes, tumor cells are disseminated by the circulation through the body during hematogenic metastasis development. Akin to leukocytes, tumor cells do not randomly leave the blood vessels, but are guided by certain signals that are also delivered by the vascular endothelium. Earliest observations on this have been documented more than hundred years ago by Paget, who regarded in his theory on breast cancer metastasis the cancer cells as a seed that can only grow at certain sites of the body, which he considered as the soil [1]. Balkwill and Mantovani worded the assumption that tumor cells might use chemokine gradients to spread around the body [2], therefore, tumor cells and leukocytes might use similar localization signals. The stromal cell-derived factor (SDF)-1α is such a chemokine, which is expressed in organs that are the first destination of breast cancer metastases [3]. We have elaborated this hypothesis on localization signals: ligands to G protein-coupled receptors (GPCRs), which are chemokines and neurotransmitters as well, might chemotactically guide tumor cells to their destination of metastasis formation [4].

The extravasation process is in principle divided into three sequential steps regardless the type of cells. In the first step, the cells slackly attach to the vascular endothelial cells. Due to this loose interaction, the extravasating cells are still pulled along with the blood stream, which results in a rolling motion of the cells on the vascular surface. In the second step, the cells tightly attach to the endothelial cells, whereas the receptors for this interaction are different from those involved in the rolling process. After attachment, the previously mostly spherical cells spread on the endothelium and actively transmigrate through the endothelial barrier. This third step of the extravasation process is called diapedesis.

In some descriptions of the extravasation process of leukocytes, two further differentiating steps are included: before the cells get in contact to the endothelium, they have to move to the fringe of the blood stream, which is termed 'margination'. There is an equilibrium between circulating and marginated leukocytes, whereas the ratio of these subpopulations changes under certain conditions [5]. At the margin of the blood vessel, the flow rate is much lower than in the centre, thus, the blood flow has a parabolic profile of variable steepness [6]. Therefore, the cells are slower at the fringe of the blood vessels, which relieves rolling and adhesion. A second additional step of the extravasation process is sometimes included between rolling and adhesion, where the leukocytes are activated by receptor-bound chemokines to attach tightly. This tight attachment is mediated by integrins, which are activated by chemokine signalling via the so-called 'inside-out' signalling. These mechanisms will be discussed in detail below together with the characterization of the rolling process, since these steps happen at the same time.

### Types of endothelium

The structure of the vascular endothelium considerably varies between organs and tissues of the body (Tab. 1). This variability consequently results in differences of the function with regard to permeability, leukocyte trafficking and homeostasis [7]. In principle, the blood vessels are constituted of a continuous/non-fenestrated (skin, lung, heart, brain) endothelial monolayer, where all endothelial cells are closely and continuously connected to each other, a continuous/fenestrated (endocrine glands, intestinal and gastric mucosa, glomeruli) endothelial monolayer, where some minor gaps occur, especially at those places, where three cells meet each other, or a discontinuous/sinusoidal (liver) endothelial monolayer, with large gaps that expose the underlying extracellular matrix (ECM) [7]. Leukocytes generally leave the blood stream in the postcapillary venules of the skin, muscle and mesentery, whereas the extravasation process is different in lung, liver and lymph nodes. In lung and liver, the cells leave the blood stream in the microvasculature, in lymphoid organs in the high endothelial venules (HEV), which possess a certain structure [7].

**Table 1:** Types of endothelium

**Tissue**	**Type of endothelium**	**Adhesion molecules**	**Site of extravasation**
**Lung**	non-fenestrated, continuous	minor role of selectins	microvasculature

**Liver**	discontinous, fenestrated	minor role of selectins, adhesion to exposed ECM	microvasculature

**Lymph organs**	HEV, plump und cuboidal	constitutive expression of L-selectin ligands	postcapillary venules, constitutive

**Skin**	non-fenestrated, continuous	adhesion molecules upregulated in case of inflammation	postcapillary venules

**Blood/brain barrier**	non-fenestrated, continuous		no regular extravasation

The pulmonary circulation differs fundamentally from the systemic circulation [8]. The concentration of leukocytes in pulmonary capillary blood is 35 to 100 fold higher than in the large vessels of systemic circulation [9,10]. As written above, extravasation occurs predominantly at postcapillary venules in the systemic circulation, whereas in pulmonary circulation extravasation takes place in the microvasculature [11,12]. Pulmonary capillaries are too small to allow rolling. Furthermore, the involvement of selectins – an important group of adhesion molecules discussed in detail below – is not clear: there are selectin-dependent and -independent mechanisms described as well [8,10]. In the liver, the hepatic artery and portal vein drain into the hepatic sinusoids; hepatic sinusoidal endothelial cells are discontinuous (6 to 8 percent of endothelium surface are fenestrae, which directly expose parts of the extracellular matrix) [13]. Next to the lung, the liver is the site in the body with the greatest part of leukocyte margination. As in the lung, the extravasation process lacks the rolling phase and endothelial cells do not express selectins, which are the predominant receptors for this process [13]. The HEV of the secondary lymph organs represent specialized postcapillary venules. A special form of leukocyte trafficking was found in these HEV of the secondary lymph organs, such as peripheral and mesenteric lymph nodes, Peyer's patches, appendix, and tonsils. Here, a constitutive transmigration of leukocytes takes place to enable leukocyte circulation between blood and lymphatic organs, in contrast to normal venules, where transmigration only occurs in case of inflammation [14,15]. Endothelial cells of the HEVs differ from their counterparts in other organs: the cells are plump or cuboidal instead of being flat [14,15].

## Rolling

### Leukocytes

Since the endothelia differ in their structural and functional features as well as in the hemodynamic pattern, there are differences in the way to recruit leukocytes [16]. In brain microvasculature, where high shear forces and low adhesion molecule expression exist, rolling seems to play a minor role. It was found that platelets function as a bridge connecting leukocytes and endothelium to mediate adhesion [16].

**Figure 1:**
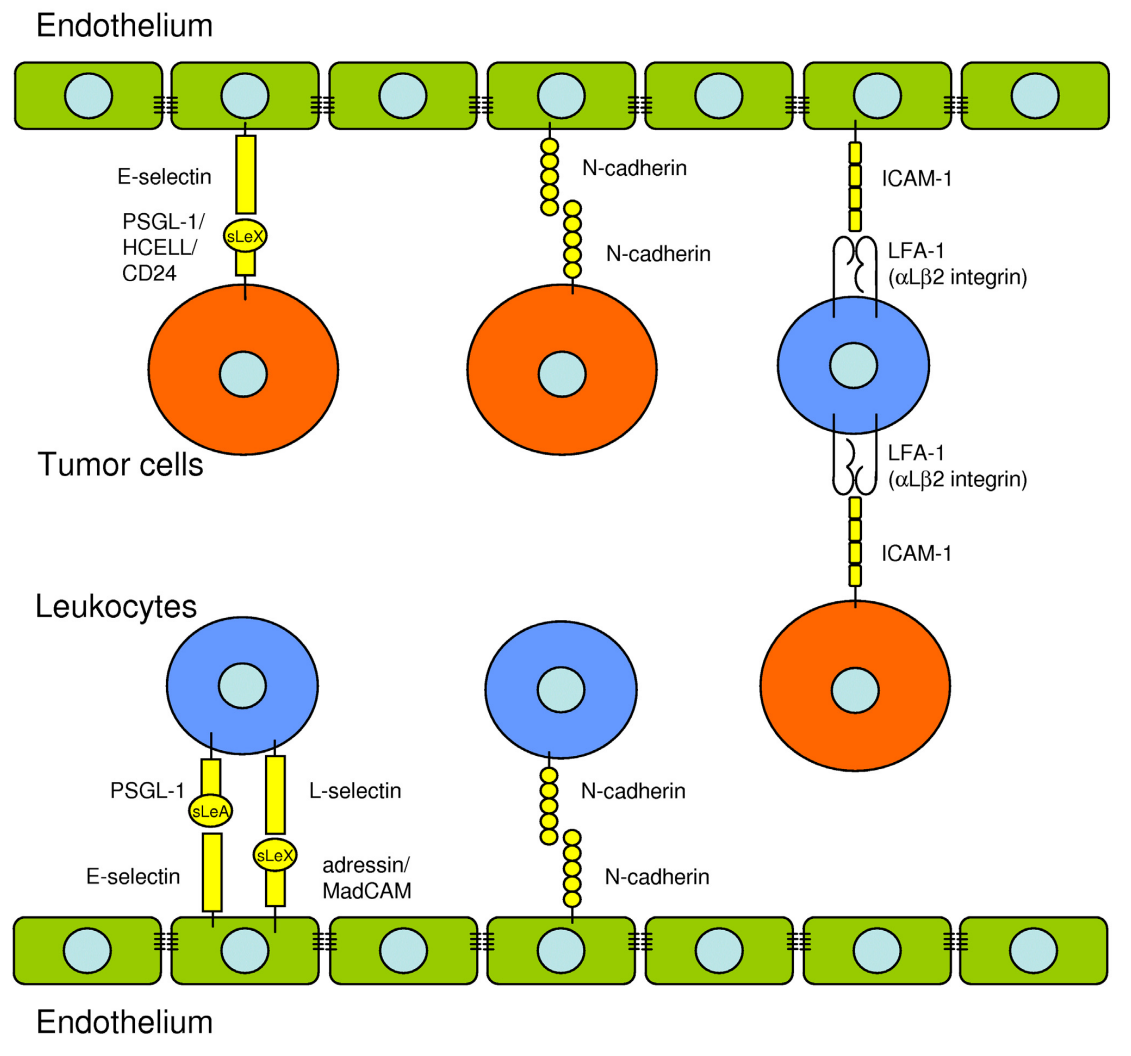
**Receptors involved in the rolling of leukocytes and tumor cells**. The endothelium is shown in green, leukocytes in blue and tumor cells in red.

Generally, rolling of leukocytes is mainly mediated by selectins, also named as lectin cell adhesion molecules, which are expressed on the surface of both endothelial cells and leukocytes (Fig. 1; Tab. 2). In contrast, tumor cells do not express selectins, but their respective ligands. The family of selectins comprises three members: the L-, P- and E-selectin (CD62L, P, E). All selectins are calcium-dependent single-chain transmembrane glycoproteins. Rolling of leukocytes on HEV is mainly mediated by L-selectin and its ligands. L-selectin ligands are different sulphated glycans, that are collectively known as peripheral lymph node adressin [17], and are constitutively expressed on HEV: GlyCAM-1 [18], CD34 [19], nepmucin [20], Sgp200 [21] and others. They can function as ligands for L-selectin when decorated with a specific carbohydrate structure known as 6-sulfo-sialyl Lewis X (sialic acidα2–3Galβ1–4 [Frucα1–3(sulfo-6)]GlcNAcβ1-R) [22]. This modification is mainly mediated by the HEV restricted N-acetylglucosamine-6-O-sulfotransferase (also known as GST-3 or LSST) [17]. LSST is constitutively expressed in HEV but recent studies have shown that it can be induced in other kinds of endothelium in case of inflammatory lesions, e.g. rheumatoid arthritis [23]. L-selectin was first defined as a homing receptor for lymphocytes, since it is a key regulator for the recruitment of naïve and central memory lymphocytes to the lymph node, a process that occurs constitutively. HEVs in mesenteric lymph nodes also express the mucosal adressin cell adhesion molecules (MadCAM) as a ligand for L-selectin [23], in order to mediate rolling and adhesion via the α4β7-integrin on leukocytes [7] (Fig. 1).

**Table 2:** Comparison of receptor-ligand pairs involved in the extravasation of tumor cells and leukocytes

	**Rolling**	**Adhesion**	**Diapedesis**
**Tumor cells**	PSGL-1 – E-selectin HCELL – E-selectin CD24 – E-selectin CEA – E/L-selectin N-cadherin – N-cadherin ICAM-1/LFA-1 mediated rolling via leukocytes	α4β1/β7 – VCAM CD44v6 – galectin-3 Lamp1/2 – galectin-3 αvβ3 – L1-CAM	N-cadherin – N-cadherin Apoptotic mechanisms

**Leukocytes**	PSGL-1 – E-selectin HCELL – E-selectin CD24 – E-selectin L-selectin – peripheral lymph node adressin L-selectin – MadCAM N-cadherin – N-cadherin	LFA-1 – ICAM-1/2 Mac-1 – ICAM-1/2 LFA-1 – JAM-A Mac-1 – JAM-C VLA-1 – VCAM L1-CAM – VLA-5 glycosylated proteins – galectins	**paracellular:** JAM-C – JAM-B LFA-1 – JAM-A VLA-4 – JAM-B Mac-1 – JAM-C CD99 – CD99 PECAM-1 – PECAM-1 **transcellular:** LFA-1 – ICAM-1 PECAM-1 – PECAM-1

In endothelium different to HEV, additionally P-and E-selectin regulate the rolling process of lymphocytes: P-selectin and L-selectin lead to rolling and initial localization of leukocytes and platelets, whereas E-selectin enhances the subsequent recruitment of leukocytes. P- and E-selectin both bind to PSGL-1 (P-selectin glycoprotein ligand-1), which is expressed on all blood lymphocytes, monocytes and neutrophils. P-selectin is preformed and stored in Weibel-Palade bodies of the endothelium and α-granules of platelets; exposure of endothelial cells to inflammatory cytokines, histamine, thrombin and other proinflammatory mediators leads to a rapid upregulation of P-selectin [24].

E-selectin is expressed within the first hours after exposure to inflammatory mediators, with the exception of the skin and bone microvessels, where it is constitutively expressed [25,26]. E-selectin ligands, e.g. CD43 and PSGL-1, are proteins that are decorated with sialyl Lewis X (sLeX) or sialyl Lewis A (sLeA). Decorated PSGL-1 is called cutanous lymphocyte antigen (CLA), that regulates skin homing of activated T cell subtypes [25]. HCELL (hematopoietic cell E-/L-selectin ligand) is a glycoform of CD44 that is expressed natively only on stem cells. It has the highest affinity of all natively expressed E-selectin ligands [27].

Furthermore, we have identified N-cadherin as a molecule involved in the rolling process of neutrophil granulocytes on pulmonary microvasculature [28]. Cadherins are calcium-dependent homophilic cell-cell adhesion molecules in any solid tissue of the body [29]. The cadherin family comprises several members with a distinct tissue expression patterns. Classical cadherins are the epithelial (E-), neural (N-), placental (P-), retinal (R-) and vascular endothelial (VE-) cadherin [29]. As the name says, N-cadherin is expressed in neural but also in other mesenchymal and connective tissues [30]. Besides N-cadherin, only the vascular endothelial (VE)-cadherin has yet been identified to play a role in the extravasation process of leukocytes, since it is expressed in endothelial adherens junctions [31]. However, it is so far not clear, how leukocytes manage crossing of that barrier [32].

### Tumor cells

Although tumor cells mimic mechanisms used by leukocytes, the adhesion molecules and ligands involved in tumor cell extravasation are somehow different suggesting additionally or alternatively non-leukocyte-like mechanisms [33]. Specific interactions between receptors of tumor cells and ligands expressed on endothelium may explain the distinct tropism of cancer cell subsets [25]. Several groups found tumor cells to express ligands for E-selectin, which leads to rolling and extravasation at endothelial sites expressing E-selectin. As written above, these are sites of inflammation or vessels of the bones, where E-selectin is expressed constitutively. The expression of these ligands correlates with a high potential of bone metastasis: HCELL, which binds to L- and E-selectin, was found on LS174 colon carcinoma cells [34], as well as further variants of CD44 that allow L-, E- and P-selectin interactions [35,36]. Hanley et al. [35] defined E-selectin mediating slow, P-selectin intermediate and L-selectin fast rolling of tumor cells. Furthermore, bone-metastatic prostate carcinoma cell lines were found to express PSGL-1 and the E-selectin ligand-1 [37]. CD24, when decorated with sLeX, also promotes hematogenic metastasis formation, but it is less efficient than PSGL-1 [38]. Recently, the carcinoembrionic antigen (CEA), which is expressed on colon carcinoma cells, has been reported to act as a mediator for rolling on E- and L-selectin, too [39].

We have shown that N-cadherin mediates the rolling of MDA-MB-468 human breast carcinoma cells on pulmonary endothelium [28]. In addition, an other group has described N-cadherin to be involved in the transmigration process [40]. In conclusion, it seems that in the case of tumor cells, there is not such a clear difference between the receptors used for rolling, adhesion and diapedesis as it is for leukocytes.

Furthermore, leukocytes facilitate the contact between tumor cells and the endothelium. Leukocytes act as bridge- or linker-cells. We have described that neutrophil granulocytes promote the adhesion of MDA-MB-468 breast carcinoma cells to pulmonary endothelium. The tumor cells lack β2-integrins, which are the ligand for the intercellular adhesion molecule (ICAM)-1 on the endothelium. Instead, the tumor cells express ICAM-1 and adhere to neutrophil granulocytes, which then act as a linker connecting the tumor cells to the endothelium and thereby enable firm adhesion [28] (Fig. 1). The same mechanism has also been described for other blood cells and other molecules involved, e.g. the interaction of platelets and leukocytes with tumor cells bearing ligands for P-selectin [41-43].

### Localization signals – role of GPCR ligands

Chemokines are ligands to GPCRs, which are known as signal substances that regulate the migratory activity of cells and chemotactically guide these cells. Chemokines are 8 to 12 kDa peptides with highly conserved structural elements. According to these elements they have been divided into four subgroups [44]. More than 50 chemokines and 20 chemokine receptors have been characterized [45]. Chemokines are soluble proteins that are presented on endothelial cells by glycosaminoglycans. These receptors are not the specific chemokine-receptors, but prevent the chemokines from rapid removal by the blood flow [22,46]. The chemokine receptors on the leukocytes are specifically expressed in certain subpopulations [47]. Therefore, leukocyte subsets are selectively susceptive to various chemokines. The signal transduction of the chemokines receptors leads to changes of the affinity and avidity of integrins on leukocytes to their specific ligands expressed on the endothelium by the so-called 'inside-out' signalling [48]. This activation of integrins is due to the phosphorylation of the cytoplasmic domain of both chains of the heterodimeric integrins [49,50], which then leads to an integrin-mediated arrest of the cells on the endothelium [51], as will be discussed in detail below.

Chemokines are functionally divided into two groups, the inflammatory and the constitutive chemokines, for they are either guiding leukocytes to sites of inflammation or to other sites such as secondary lymph organs. For example, CCL17/TARC (thymus- and activation-regulated chemokine) functions as a skin-homing factor. It is presented on the skin microvasculature and induces the adhesion of CCR4 bearing CD4^+ ^lymphocytes [52]. HEV express constitutively CCL21, which activates the leukocyte function-associated antigen (LFA)-1 by a G protein-mediated pathway. LFA-1 is the αLβ2-integrin, on rolling CCR7 expressing lymphocytes. Consequently, this leads solely to the adhesion of these cells and no other leukocyte subsets transmigrate through the HEV [7,22,53]. SDF-1α (CXCL12) is described as a very potent constitutive chemokine for the localization of lymphocytes, homing of stem cells to bone marrow and tumor cells [54]. SDF-1α was the first chemokine identified to play a role in the localization of breast cancer metastases [3]. Meanwhile, it is generally accepted that similar to leukocytes, tumor cells use chemokines as guidance signals, too [55-57]. SDF-1α plays also a role in B16 melanoma cell adhesion via integrins [58], and CX3CL1 (fractalkine) has been reported to guide prostate carcinoma cells to the bone marrow endothelium, which constitutively expresses this chemokine [59,60].

Besides chemokines, we have suggested that neurotransmitters play a role in the localization of metastases, too [4]. Like chemokines, neurotransmitters mostly bind to GPCRs. However, there is no common neurotransmitter receptor, which could present neurotransmitters on the endothelial surface. But neurotransmitters provide strong chemotactic signals for tumor cells [61,62], and might indirectly deliver localization signals by stimulating the release of such signals from the endothelial cells [63]. We have shown that the development of lymph node metastases of PC-3 prostate carcinoma cells in mice increases by norepinephrine treatment [64].

## Adhesion

### Leukocytes

Integrins are the main adhesion molecules of leukocytes. During the rolling step, integrins are activated, and then bind to their counterparts on the endothelial cells to mediate a tight attachment (or arrest) of the leukocytes. Integrins are single transmembrane cell adhesion molecules consisting of two non-covalently associated α and β chains. In vertebrates 18 α and 8 β subunits form 24 known αβ dimmers, whereas β2 and β7 integrins are exclusively expressed on leukocytes [65]. Chemokines are the most powerful physiological activators of integrin-mediated adhesion. Within less than a second they regulate integrin affinity and avidity in a cell-type specific manner by inside-out signalling [66,67]. This signalling induces integrins to undergo conformational changes from a bent low-affinity state to an extended intermediate- or high-affinity state resulting in the opening of the ligand-binding pocket [68]. However, chemokine-stimulated inside-out signalling induces conformational changes in only a fraction of integrins [51]. The intracellular signalling cascades leading from GPCR activation to integrin activation are still incompletely understood, since different cell types, species and integrins use different signalling steps. A linear signal-transduction pathway would be a simplification of the complex network between chemoattractant-binding and integrin-adhesion [69]. Key regulatory events are the phosphorylation of the cytoplasmic domains [49,50], as well as binding of talin [70].

**Figure 2:**
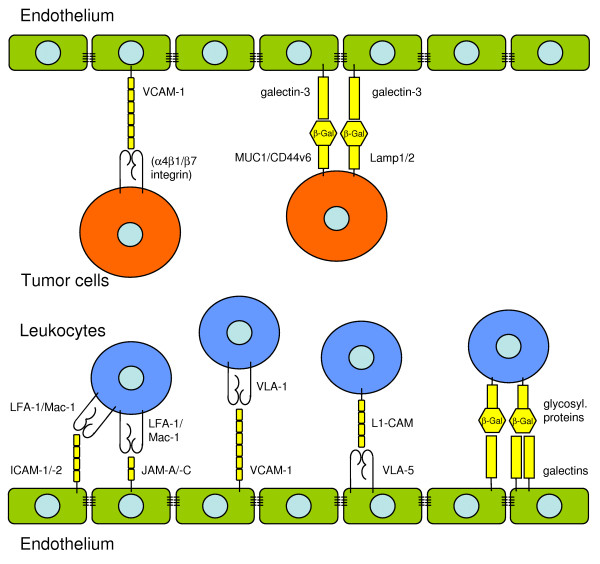
**Receptors involved in the adhesion of leukocytes and tumor cells to endothelial cells**. The endothelium is shown in green, leukocytes in blue and tumor cells in red.

The integrin receptor family is subdivided into 8 groups according to the β chains. The following groups are most widely studied: β1 (CD29) = very late activation antigen (VLA) integrins, which mediate mainly cell-matrix interactions; β2 (CD18) = leukocyte integrins, which mediate mainly cell-cell interactions; β3 (CD61) with various adhesive functions, and β7 integrins mediating cell-cell interactions (Tab. 2). Those integrins that are most important in leukocyte arrest belong to the β1 and β2 subfamilies [33,51]: the αLβ2 (lymphocyte function-associated antigen (LFA)-1) and αMβ2 (Mac-1) are both ligands for ICAM-1/-2 (Fig. 2), whereas αLβ2 can also bind to the junctional adhesion molecule-A (JAM-A), and αMβ2 to JAM-C on the endothelium, which will be explained in detail in the diapedesis section. The α4β1 integrin VLA-4 is the ligand for the vascular cell adhesion molecule (VCAM)-1. Neutrophil granulocytes express VLA-4 only in case of sepsis [71]. ICAM-1/-2 (CD54/CD102) are expressed on surface of resting endothelium at low levels, but ICAM-1 becomes upregulated at sites of inflammation, e.g. in response to the tumor necrosis factor (TNF)α or interleukin (IL)-1β [65]. VCAM-1 (CD106) is also upregulated in response to inflammatory mediators. VCAM-1 is described as a key molecule in monocyte recruitment to early lesions in experimental models of atherogenesis [72,73]. Thus, on the side of the endothelium, several members of the immunoglobuline (Ig) superfamily are involved in the adhesion of leukocytes [74]. Furthermore, integrins that are expressed on endothelial cells are involved in leukocyte adhesion, too. The integrins α5β1 (VLA-5) and αvβ3 on the endothelium bind to L1-CAM, which is a molecule of the Ig superfamily expressed on lymphocytes [33,75,76].

There is a growing body of evidence, that galectins are involved this part of the extravasation. Galectins are defined by their ability to recognize β-galactose because of their conserved carbohydrate-recognition domains (CRDs) of about 130 amino acids. Fifteen mammalian galectins have been identified, which can be subdivided into three groups: those with one CRD, those with two CRDs, and chimera galectins with a tail of short tandem repeats fused to one single CRD [77,78]. Galectins can be secreted or, depending on the cell-type and activation state, be localized in the nucleus, the cytoplasm or the cell surface, where they interact with appropriately glycosylated proteins [79]. The most extensively studied function of galectins is their involvement in regulation of apoptosis (galectin-3 has anti-apoptotic function, galectin-1 is a pro-apoptotic receptor) [80,81], but during the past ten years it became evident that galectins are modulators of the immune response (pro- and anti-inflammatory functions) [82], as well as modulators of cell migration, adhesion and angiogenesis [78].

Galectin-1 is a non-covalent homodimer of two single CDR molecules, expressed in cultured endothelia of different origin as human aorta, umbilical vein, pulmonary artery, and mouse liver, brain and lung [78]. The activation of endothelial cells, e.g. with lipopolysaccharides, or tumor-conditioned media results in a rapid increase of galectin-1 expression, and it is constitutively expressed in tumor-associated endothelial cells [83,84]. Galectin-1 has anti-inflammatory properties. Peretti et al. observed that endogenous galectin-1 inhibits the adhesion of leukocytes to the endothelium, in the case of neutrophil granulocytes this was due to a suppression of Mac-1 upregulation [85-87]. Thus, galectin-1 seems more to function as a signalling molecule in the regulation of integrins than to act as a classical adhesion molecule. Accordingly, the upregulation of galectin-1 in tumor-associated endothelial cells is postulated as a tumor escape mechanism from immune response [88].

The expression of galectin-3 on endothelial cells is reported in vitro in human umbilical vein and dermal microvasculature as well as mouse lung, brain and liver [78]. Galectin-3 is upregulated in endothelial cells of cancer tissue as found in vivo and in a murine tumor model of hepatocellular carcinoma [89]. Nieminen et al. recently found a role for galectin-3 in the β2 integrin-independent adhesion of neutrophil granulocytes to lung endothelium in case of *Streptococcus *infection [90].

Galectin-9 was originally identified as a chemoattractant for eosinophil granulocytes [91]. Recently, it was identified as a receptor for the T_helper1 _lymphocyte-specific cell-surface molecule Tim-3, thus negatively regulating T_helper1 _lymphocyte responses [92]. Galectin-9 is expressed in various endothelial cells but so far mainly studied in the context of viral infections.

### Tumor cells

As mentioned above, the expression of several integrins, especially of the β2 subgroup, is restricted to leukocytes. Thus, tumor cells must use some different receptors or mechanisms for their adhesion to the endothelium, such as utilizing leukocytes as linker cells (Fig. 1). However, expression of the α4 integrin was demonstrated on many different human tumors and tumor cell lines, especially on melanoma and sarcoma. Together with the β1 or β7 integrin, the α4 integrin can act as a ligand for VCAM-1 and fibronectin. Animal models demonstrated, that the metastatic capacity of melanoma cells enhanced through α4β1 integrin interactions with VCAM-1 [93,94]. Furthermore, expression of the αvβ3 integrin has been associated with the metastatic potential of tumor cells, but it seems more to be involved in transmigration by interacting with L1-CAM, than in the adhesion process [95]. Thus, as already mentioned for the role of N-cadherin in tumor cell rolling and diapedesis, the receptor involvement in each step of the extravasation process in tumor cells is yet not as clearly defined as in leukocytes.

In contrast to integrins, galectins are a group of adhesion receptors that leukocytes share with tumor cells. In several experimental systems galectin-3 expression in cancer cells was associated with a metastatic phenotype [96]. During the last twenty years it became evident, that an altered glycosylation and exposure of glycoproteins by tumor cells may facilitate their adhesion to endothelial galectins [78]. The Thomsen-Friedenreich antigen (TA) is expressed on human breast and prostate carcinoma cell lines and serves as a ligand for galectin-3 expressed on endothelium, leading to increased tumor cell adhesion [97-99]. TA is a simple mucin-type disaccharide with β-galactose as a terminal sugar (Galβ1-3GalNAc). It is expressed on the surface of T cell-lymphomas and most human carcinomas [100,101], so far two surface molecules are known to represent TA: MUC1 [102] and CD44v6 [103]. Furthermore, lysosomal-membrane-associated glycoproteins (Lamps) 1 and 2 are described as potential binding partners for galectin-3. Lamps are rarely found on the plasma membrane of normal cells, but there is an increased surface expression in tumor cells [104].

Clausse et al. observed that culture media, that were conditioned by incubation with the prostate carcinoma cell line PC-3, induced galectin-1 expression on the surface of endothelial cells. This resulted in an increased attachment of the tumor cells. The effect was inhibited by the addition of a galectin-1-specific antibody [83]. This stands in clear contrast to the above discussed role of galectin-1 in leukocyte adhesion: here, adhesion was decreased in case of galectin-1 expression.

## Diapedesis

Leukocytes transmigrate through the endothelium without irreversibly impairing its integrity. There are two routes that the cells can use and it is not clear, to which part each of these ways is involved in the diapedesis overall. The leukocytes can either move between the endothelial cells, which is termed the paracellular or junctional route, or they can migrate through an endothelial cell, which is termed the transcellular route. Which way the leukocytes take, obviously depends on several circumstances. Leukocytes subpopulations prefer different routes, and the decision seems to depend on further factors, e.g. the duration of endothelium activation with cytokines [105].

### Paracellular route

**Figure 3:**
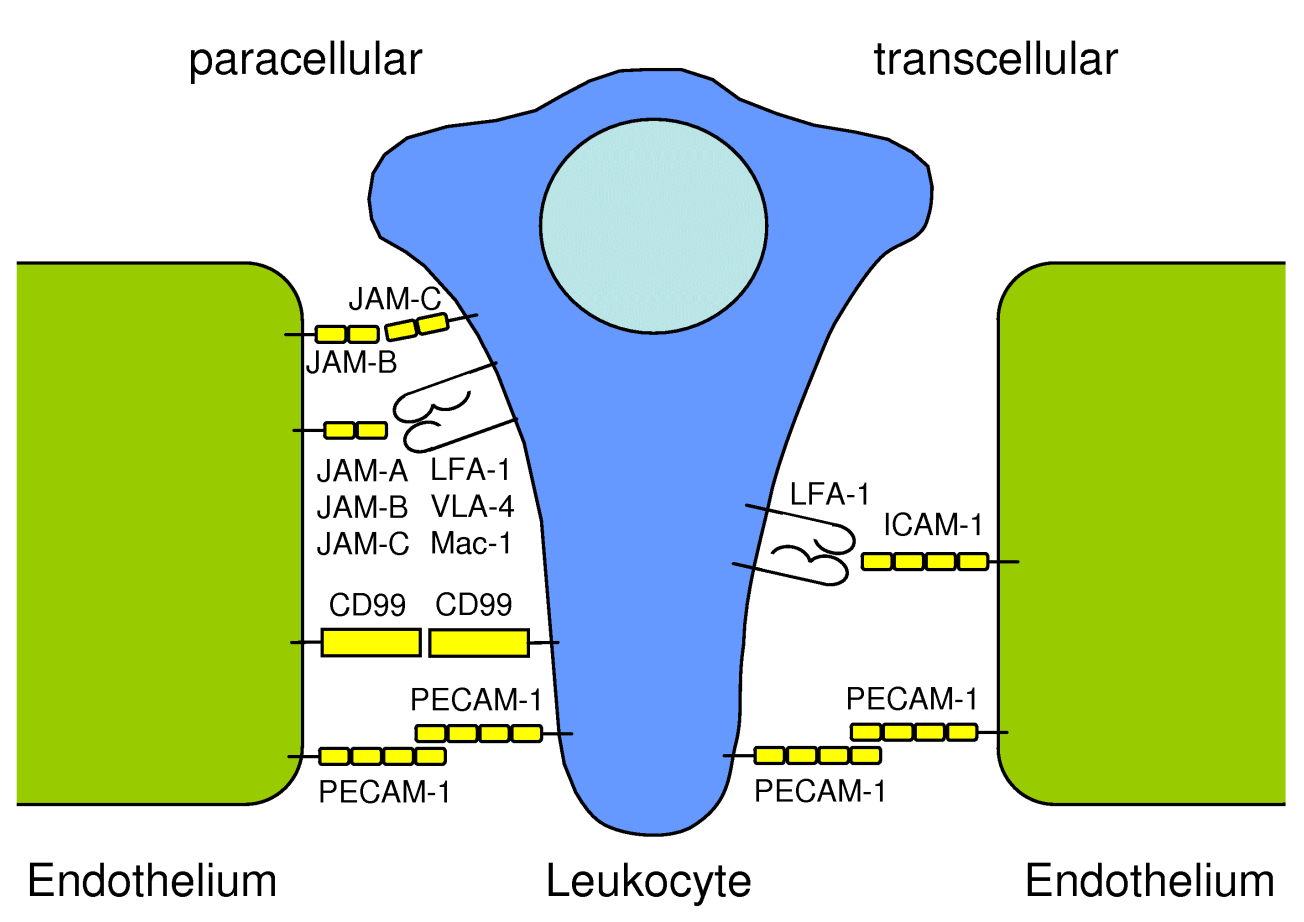
**Transmigration of leukocytes through endothelium**. Receptors involved in the paracellular or junctional route are shown on the left side, receptors involved in the transcellular route are shown on the right side. The endothelial cells are shown in green, the leukocytes in blue.

When leukocytes transmigrate between two endothelial cells, they have to pass tight junctions and adherens junctions. Therefore, the connection between the endothelial cells has to be temporarily abrogated and substituted by the corresponding binding molecule on the leukocyte surface. The platelet/endothelial cell adhesion molecule-1 (PECAM-1; CD31) is one of these molecules (Fig. 3). It is a member of the Ig superfamily, homophilic binding, and expressed on vascular endothelial cells as well as leukocytes. It is one of the most important adhesion molecules for the diapedesis of leukocytes. Blockade of this receptor inhibits the transmigration of neutrophil granulocytes [106], monocytes [106,107], and NK cells [108]. In contrast, CD31 is dispensable for the transmigration of lymphocytes [109]. However, there are reports showing that PECAM-1 has to be regarded not only as an adhesion receptor but also as a signalling receptor [110].

CD99 and the related molecule CD99L2 are further adhesion molecules in the transmigration of leukocytes. CD99 is involved in monocyte, neutrophil granulocyte and lymphocyte diapedesis [111-113], but CD99L2 is not relevant for lymphocyte diapedesis to inflamed tissue [112]. Like PECAM-1, it is expressed on both leukocytes and endothelial cells, and it has homophilic binding properties [105,110].

A third group of receptors that have been identified to play a role in the paracellular diapedesis of leukocytes are the junctional adhesion molecules [114]. JAMs belong to the Ig superfamily having two extracellular Ig domains. To date, three members are known, designated as JAM-A to -C (or JAM-1 to -3), whereas JAM-A is best characterized for its role in leukocyte diapedesis. Initially, JAM-A has been characterized as a homophilic adhesion receptor of tight junctions on epithelial and endothelial cells [105,115]. Furthermore, JAM-A binds to LFA-1 (αLβ2 integrin) [116]. JAM-B binds to VLA-4 (α4β1 integrin), and JAM-C binds to Mac-1, (αMβ2 integrin). All of the integrins are expressed on leukocytes and consequently, endothelial JAMs are supposed to play a crucial role in leukocyte diapedesis [105,110,117]. It is worth of note, that JAM-C is also expressed on leukocytes and interacts with JAM-B on endothelial cells [118]. The endothelial selective adhesion molecule (ESAM) is a receptor related to JAMs. However, its expression is restricted to endothelial cells and activated platelets, no binding partner or counter-receptor on leukocytes has been identified so far [110].

### Transcellular diapedesis

As compared to the paracellular route, much less is known about the transcellular diapedesis. This way of extravasation seems to be less used but much faster than the paracellular transmigration, whereas the part and kind of leukocytes using this route varies obviously in dependence on the type of endothelium [51,105]. ICAM-1 on the endothelial cells is best characterized to play an important role in the transcellular migration. ICAM-1 binds to the integrin LFA-1 on leukocytes. ICAM-1 is redistributed to caveolae-rich regions, where vesiculo-vacuolar organelles form intracellular channels [51]. Besides ICAM-1, PECAM-1 has been identified to be involved in this extravasation route, and in general, the assumption has been made that the same molecules that mediate paracellular migration are mediators for the transcellular migration, too [117].

When the extravasating cells have successfully passed the cellular barrier, they face a further hurdle. This is the basement membrane, to which the leukocytes in fenestrated or discontinuous endothelia, e.g. of the liver, can also attach directly via integrin receptors to matric components. In order to cross this barrier, leukocytes express matrix-metalloproteinases (MMPs), e.g. T lymphocytes express gelatinases A and B, which are two MMPs that digest the major basal lamina constituents collagen types IV and V [119]. However, it is not clear, if MMPs only play a role in the extravasation process or whether they contribute to the migration of leukocytes through tissue [120].

### Cancer cell diapedesis

In contrast to leukocytes, tumor cells are supposed not to leave the endothelium intact after diapedesis. This is understandable just from the bare size of the cells. Tumor cells are much larger than leukocytes and it would be difficult to squeeze between endothelial cells without any damage. One of the few reports on the mechanisms of tumor cell diapedesis aims on the influence of N-cadherin [40], which we have identified to mediate rolling of tumor cells, too [28].

Consequently, a new model completely skips the transmigration process: metastatic tumor cells attach to the endothelium of pulmonary capillaries. Transmigrated cells were cleared quickly by the immune cells of the lung, whereas early tumor colonies were entirely attached to endothelium on the intravascular side, as investigated by intact organ epifluorescence microscopy in mice [121]. However, we have observed in an in vitro model, that T24 bladder carcinoma cells transmigrate through a monolayer of human umbilical vein endothelial cells (HUVECs). During transmigration, the endothelium is irreversibly damaged [122], which is probably due to induction of apoptosis by the loss of cell-cell contacts [123]. Such an endothelial retraction is currently the favored model for tumor cell diapedesis [33].

In conclusion, the first two steps of the extravasation of tumor cells and leukocytes, rolling and adhesion, seem to have similarities with regards to the mechanisms and receptors involved. In contrast, the third step, the diapedesis, is fundamentally different between these two cell types, whereas there is somehow a lack of knowledge with regard to tumor cells. But it seems that the function of leukocytes as guardians of the body is reflected in a cautious manipulation of the endothelial cells. Tumor cells on the contrary display a destructive behaviour.

## List of abbreviations

CEA: carcinoembrionic antigen; CLA: cutanous lymphocyte antigen; CRD: carbohydrate-recognition domains; ECM: extracellular matrix; GPCR: G protein-coupled receptor; HCELL: hematopoietic cell E-/L-selectin ligand; HEV: high endothelial venules; HUVEC: human umbilical vein endothelial cells; ICAM: intercellular adhesion molecule; Ig: immunoglobuline; IL: interleukin; JAM: junctional adhesion molecule; LFA: leukocyte function-associated antigen; MadCAM: mucosal adressin cell adhesion molecule; MMP: matrix-metalloproteinase; NK cells: natural killer cells; PECAM: platelet/endothelial cell adhesion molecule; PSGL: P-selectin glycoprotein ligand; SDF: stromal cell-derived factor; TA: Thomsen-Friedenreich antigen; TARC: thymus- and activation-regulated chemokines; TNF: tumor necrosis factor; VCAM: vascular cell adhesion molecule; VLA: very late activation antigen.

## Competing interests

The authors declare that they have no competing interests.

## Authors' contributions

Both authors together wrote this manuscript.
